# The Role of Vitamin D Binding Protein, Total and Free 25-Hydroxyvitamin D in Diabetes

**DOI:** 10.3389/fendo.2019.00079

**Published:** 2019-02-19

**Authors:** Rolf Jorde

**Affiliations:** ^1^Tromsø Endocrine Research Group, Department of Clinical Medicine, UiT The Arctic University of Norway, Tromsø, Norway; ^2^Division of Internal Medicine, University Hospital of North Norway, Tromsø, Norway

**Keywords:** diabetes, free vitamin D, vitamin D binding protein (DBP), single nucelotide polymorphisms, 25- hydroxy vitamin D

## Abstract

Vitamin D is important for bone health, but may also have extra-skeletal effects. Vitamin D and its binding protein DBP have immunological effects and may therefore be important in the development of type 1 diabetes (T1DM), and low serum levels of 25-hydroxyvitamin D (25(OH)D) are associated with later development of type 2 diabetes (T2DM). However, it has so far been difficult to convincingly show an effect of vitamin D supplementation on prevention or treatment of diabetes. The serum level of 25(OH)D has traditionally been used as a marker of a subject's vitamin D status. This measurement includes both 25(OH)D bound to DBP and albumin as well as the free from of 25(OH)D. However, according to the free hormone hypothesis, the free form is the biologically active. Previously the free form of 25(OH)D had to be calculated based on measurements of 25(OH)D, DBP, and albumin, but recently a method for direct measurement of free 25(OH)D has become commercially available. This is important in clinical conditions where the amount of DBP is affected, and has caused a renewed interest in which vitamin D metabolite to measure in clinical situations. In the present review the relations between DBP, total and free 25(OH)D in T1DM and T2DM are described.

## Introduction

Vitamin D is produced in the skin upon UV-B exposure and is obtained through the diet where fatty fish is the main source. Regardless of how it is obtained, vitamin D has to be hydroxylated first in the liver to 25-hydroxyvitamin D (25(OH)D) and then in the kidneys to the active form 1,25-dihydroxyvitamin D (1,25(OH)_2_D) (1). These hydroxylations may also occur in peripheral tissues (2).

In the circulation the major part of vitamin D, 25(OH)D and 1,25(OH)_2_D are bound to the vitamin D binding protein (DBP), and to a lesser extent also to albumin. Only a small fraction circulates in the free form (3). To exert their action, the vitamin D metabolites have to cross the cell membrane into the cell [and for vitamin D and 25(OH)D also to be hydroxylated], where the active form 1,25(OH)_2_D connects to the nuclear vitamin D receptor (VDR) (1).

The endocytic receptors megalin and cubulin are present in the renal tubuli and parathyroid cells (4), and at least in the kidney enable transportation of the DBP-vitamin D complexes into the cells (5). In other (and perhaps most) cell types, the vitamin D metabolites have to pass the cell membranes in their free un-bound form by passive diffusion (6).

The serum concentrations of vitamin D and 25(OH)D are >100 times that of 1,25(OH)_2_D, and the DBP binding coefficients as well as the potential for passive diffusion through cell membranes differ between these vitamin D metabolites (6). Accordingly, it is difficult to say which vitamin D metabolite, or vitamin D metabolite-DBP complex is quantitatively the most important for VDR activation and the one that should be measured for evaluation of a subject's vitamin D status (6, 7).

For this purpose, one has traditionally measured the serum 25(OH)D level, since this metabolite is abundant, easy to measure, and has a long half-life and therefore stable levels. Furthermore, the hydroxylation from vitamin D to 25(OH)D is substrate driven and the serum 25(OH)D level correlates strongly with sun exposure and vitamin D intake and also correlates with known vitamin D effects, like the suppression of the parathyroid hormone (PTH) secretion (1).

The serum 25(OH)D that is measured is the total 25(OH)D, which includes the DBP and albumin bound 25(OH)D as well as the free form. Since the major part of 25(OH)D is bound to DBP, the concentration of total 25(OH)D will depend on the serum DBP concentration. The DBP concentration is fairly stable throughout life, but increases with pregnancy and estrogen supplementation. DBP is synthesized in the liver and accordingly the serum DBP concentration is reduced in liver failure as well as in malnutrition (8, 9). Loss of proteins in the urine (like in some subjects with diabetes) may also cause low serum DBP levels (10, 11). Thus, in situations with high serum DBP levels like pregnancy, an even larger portion of the total 25(OH)D in plasma is bound to DBP and accordingly the free form is reduced. Conversely, in patients with liver cirrhosis where the serum level of DBP is low, the free fraction is increased. Although there is a strong correlation between total and free 25(OH)D (12), measurement of total 25(OH)D may therefore not always reflect the free form.

According to the free hormone hypothesis, it is the free form of the hormone, which easily diffuses through cell membranes, that is the biologically active, and the one to be measured (13). This is exemplified for thyroid hormones, where the serum concentration of tree thyroxine is regulated in a negative feedback manner by the secretion of thyroid stimulating hormone (TSH). In this system, changes in the concentration of thyroid hormone binding globulin (TBG) will be compensated by increased or decreased secretion of TSH keeping the free concentration of thyroxine stable (14). This demonstrates the utility of the free hormone concept for thyroid hormones.

This concept does not necessarily apply to the vitamin D system where the active hormone 1,25(OH)_2_D can be transported into (at least some) cells in a DBP-complex, and also have its activating hydroxylations intracellularly. Furthermore, 25(OH)D is in essence a pro-hormone not regulated by negative feed-back control. Changes in DBP will not induce changes in the hydroxylation of vitamin D to 25(OH)D since this is a substrate driven process. Increased serum 25(OH)D concentrations may be accompanied by an increased level of FGF-23, increased *CYP24A1* expression and 24-hydroxylase activity, and accelerated degradation of 25(OH)D to 24,25(OH)_2_D (15). However, this mechanism must for 25(OH)D be of minor importance since the increase in free 25(OH)D and total 25(OH)D after vitamin D supplementation is, at least until serum 25(OH)D levels of approximately 150 nmol/L, quite linear (12). Therefore, whether the total or the free form of 25(OH)D is the best vitamin D parameter cannot be decided on theoretical grounds only, but has to be tested in clinical situations as well (16, 17).

There are many single nucleotide polymorphisms (SNPs) in the *DBP* gene (*GC* gene, globulin–complex gene). Combinations of two of these (rs7041 and rs4588) result in three polymorphic alleles and six major phenotypes. These phenotypes may have different binding affinities for the vitamin D metabolites (18) and the serum 25(OH)D levels do differ between subjects with different DBP phenotypes (12). The distribution of the six variants also differs between races (19).

In addition to the skeleton vitamin D deficiency has been associated with a number of diseases, like mortality, cancer, immunological diseases, cardio-vascular diseases, and diabetes (20). Most of these relations are based on observational studies only, where 25(OH)D has been measured in old serum samples and subsequent diseases recorded. For these studies, measurement of total serum 25(OH)D has been employed, whereas there has been little focus on DBP [where the major part of the circulating 25(OH)D is bound] or the free form which potentially may be the most important.

The serum level of free 25(OH)D has traditionally been calculated based on measurements of total 25(OH)D, DBP, and albumin concentrations (21–23). However, measurement of DBP depends on type of antibody employed (monoclonal or polyclonal) (19), and it has usually been assumed that the vitamin D binding-coefficient for each of the six prevalent DBP phenotypes are equal. The validity of the free 25(OH)D calculations have therefore been questioned (24). Lately, kits for direct measurement free 25(OH)D has become commercially available which has caused a renewed interest in the relation between free serum 25(OH)D, as well as DBP, and disease states (25). However, further validation and standardization of this assay is still needed in subjects with major illnesses or with abnormal DBP or protein concentrations (16).

In the present review these relations will be summarized for the metabolic disorders type 1 and type 2 diabetes (T1DM and T2DM), presented separately.

## T2DM

### Serum 25(OH)D and T2DM

There are many reasons for why vitamin D could influence the development of T2DM. Thus, the vitamin D activating hydroxylases and the VDR are found in the pancreatic beta-cells (26, 27), 1,25(OH)_2_D may induce insulin secretion (28), and vitamin D may have an anti-inflammatory effect that may prevent insulin resistance (29).

In line with this, there are a number of observational studies on the relation between serum 25(OH)D concentration and incident diabetes, and practically all confirm an association (30). Thus, in a study by Afzal et al. on 31,040 subjects with measurement of serum 25(OH)D followed for up to 34 years, participants who had a 20 nmol/L reduction in 25(OH)D had a 16% increased risk of T2DM (31). Similarly, Ye et al. combined 22 studies in a meta-analysis that included 8,492 cases and 89,698 controls and found a 21% increased risk of T2DM per 25 nmol/L lower 25(OH)D concentration (32).

However, for vitamin D there is a strong possibility of revers causation and other methods than observational studies are needed for confirmation, as recently reviewed by Angelotti and Pittas (30).

There are a few RCTs with vitamin D specifically designed for prevention of T2DM in subjects at risk. Thus, Davidson et al. included 109 subjects with prediabetes and randomized them to high dose vitamin D (mean weekly dose 88,865 IU) vs. placebo. However, no significant effects on insulin secretion, insulin sensitivity or development of diabetes were found after 1 year (33). Similarly, in a study from Tromsø, Norway, Jorde et al. randomized 511 subjects with reduced glucose tolerance to 20,000 IU vitamin D per week vs. placebo for a maximum of 5 years, but found no difference between the groups in development of T2DM (34). However, both studies were underpowered for detection of minor effects. And finally, the effect of giving vitamin D to subjects with established T2DM do at best show a marginal effect on HbA_1c_ with a reduction of 0.32% in HbA_1c_ as compared with placebo according to a review by Lee et al. that included nine trial with 3,324 participants (35).

Another approach to the vitamin D—T2DM question is the Mendelian randomization. Several SNPs are associated with serum 25(OH)D level; SNPs in the *DHCR7* gene related to vitamin D synthesis, the *CYP2R1* gene related to 25-hydroxylation, and the *CYP24A1* gene related to 24-hydroxylation and degradation (36). When these SNPs are combined to a genetic score, the highest vs. the lowest scores result in 5–20% difference in serum 25(OH)D levels. However, in the most recent and largest meta-analysis including five studies with 28,144 cases and 76,344 non-cases, no significant association with T2DM was found, neither for the individual SNPs tested, nor when combined to a genetic score (32).

There are, however, many shortcomings of the Mendelian randomization approach. So far it only predicts differences in serum 25(OH)D concentration and not the free fraction, and the alleles tested only explain a small part of the variance in serum 25(OH)D level.

One may therefore conclude that although a low serum 25(OH)D level do predict development of T2DM, this is most likely due to confounding or reverse causality, although minor effects cannot be excluded. Hopefully the ongoing D2d study that has included 2,423 participants with prediabets randomized to 4000 IU vitamin D daily vs. placebo may settle this question (37).

### Free 25(OH)D and T2DM

There are several reports where the directly measured free fraction of 25(OH)D has been compared with total 25(OH)D regarding biological effects of vitamin D. Thus, Johnsen et al. found a better correlation for free than for total 25(OH)D regarding bone density (24), whereas that was not found in study by Michaelsson et al. (38). For PTH similar relations have been found for free and total 25(OH)D in most studies (24, 39–41), whereas Lopez-Molina et al. in healthy children found better correlation with markers of phosphocalcic metabolism for free than for total 25(OH)D (42). Shieh et al. found in the early phase (first 4 weeks) of vitamin D treatment the free 25(OH)D, but not the total 25(OH)D, to be associated with a decrease in serum PTH (43). In inflammatory diseases the results are also mixed with free 25(OH)D correlating better to disease activity in ulcerative colitis (44), whereas total 25(OH)D correlates best to activity in systemic lupus erythematosus (45). For markers of inflammation (IL-6) in older men free and total 25(OH)D appear to correlate equally (46). And finally and most important, in a study by Yu et al. the free but not total 25(OH)D was associated with risk of mortality in patients with coronary artery disease (47). The study included 1,387 patients followed for a median time of 6.7 years, during which period 205 patients died. The all-cause mortality was 64% higher in the lowest free 25(OH)D quartile vs. the highest free 25(OH)D quartile, whereas the corresponding analysis using 25(OH)D did not show a significant difference or trend across the quartiles.

So far, there are no studies where the free 25(OH)D has been compared with total 25(OH)D as predictor for development of T2DM. However, there is a publication by Lee et al. that included 1,189 non-diabetic subjects where the free and total form of 25(OH)D were measured and related to acute insulin response and glucose disposition index based on intravenous glucose tolerance tests (48). Both free and total 25(OH)D were positively associated with these measures, but after adjustment for BMI, only free 25(OH)D was significant related to insulin secretion.

Based on the above papers, one cannot conclude that measurements of free vs. total serum 25(OH)D has any advantage regarding vitamin D responses. This is also difficult to decide, as comparisons of *P*-values and correlation coefficients give indications only.

### DBP and T2DM

In addition to being the carrier protein for vitamin D and its metabolites, DBP has a number of other effects. It acts as a carrier for free fatty acids (49), it binds actin and may prevent actin polymerization during tissue damage (50, 51), may act as a macrophage activator and play a part in the inflammation process by influencing the T-cell response (52). These immunological effects may differ between the phenotypes (53), and the level of DBP as well as the different DBP phenotypes might therefore at least theoretically affect the development of not only T1DM (see later) but also T2DM.

However, in a case-cohort study design with 958 cases and 3,489 controls Jorde et al. found no association between DBP phenotypes (based on genotyping of rs4588 and rs7041) and incident T2DM (54). Furthermore, there were no relations between the DBP phenotypes and lipids and blood pressure, but a slight relation to hip circumference.

Prior to our study Wang et al. made a meta-analysis on *DBP* SNPs and T2DM that included six studies (three Caucasian and three Asian cohorts) with 1,191 cases and 882 controls. No overall association between the *DBP* SNPs rs4588 and rs7041 and T2DM was found. However, when analyzing the Asian cohorts separately, there were significant associations with T2DM for both rs7041 and rs4588 (55).

Also after the meta-analysis by Wang et al., Ye at al. meta-analyzed the *DBP* SNP rs4588 regarding T2DM in European cohorts including 28,144 cases and 76,344 controls. A strong relation between rs4588 and serum 25(OH)D was found, but not with T2DM (OR 1.00 (CI, 0.97 −1.03) (32). Accordingly, at least in Caucasians there appears to be no relation between DBP phenotypes and development of diabetes.

To the author's knowledge, there are no longitudinal studies regarding serum levels of DBP and T2DM. However, there is one cross-sectional study by Leong et al. on 2,122 adult subjects that included 201 with diabetes (56). The effect estimate per 50 mg/L DBP increase was 0.79 (95% CI 0.65–0.96) for diabetes, and there was a marginal relation between higher DBP and lower fasting blood glucose levels. However, as a cross-sectional study it could not examine the impact of biological variability of DBP over time.

## T1DM

### Serum 25(OH)D and T1DM

The 1α-hydroxylase, necessary for activation of vitamin D, is expressed in immune cells like the B- and T-cells and the antigen presenting cells (2). These cells may therefore synthesize active vitamin D locally. Vitamin D has immune-modulatory effects (57), and since T1DM is an autoimmune disorder, a role for vitamin D in pathogenesis as well as treatment thus possible (58).

However, in a study by Thorsen et al. using a case-cohort design that included 459 children with T1DM and a control group of 1,561, no association between maternal serum 25(OH)D levels sampled repeatedly during pregnancy and subsequent T1DM in the offsprings was found (59). Furthermore, in two large Danish populations, one case-cohort study with 912 cases and 2,866 controls followed for a maximum of 31 years and a case-control study with 527 matched pairs followed for a maximum of 23 years, Jacobsen et al. found no relation between neonatal vitamin D status and later risk of T1DM (60). On the other hand, there might be a link between intake of vitamin D in childhood and development of T1DM as reported by Hyppönen et al. in a birth-cohort study with 12,055 pregnant women in northern Finland (61). This was also the conclusion in a meta-analysis by Dong et al. from 2013 that included eight studies (six case-control and two cohort studies) with vitamin D intake during early life where the pooled OR for T1DM was 0.71 (95% CI, 0.51–0.98) (62).

Furthermore, the serum levels of 25(OH)D are lower in subjects with newly diagnosed T1DM (63) as well as later in the course of disease compared to similarly aged subjects (64). There may also be a beneficial effect by vitamin D supplementation in newly diagnosed T1DM. This was reviewed by Gregoriou et al. (65) who found a positive effects on the daily insulin dose, fasting, and stimulated C-peptide response by vitamin D in two studies. However, only 67 patients were randomized and the effect was marginal.

To the author's knowledge there are no studies reporting free 25(OH)D levels in T1DM.

## DBP and T1DM

Since the immunological effects of DBP may differ between the DBP phenotypes (53), relations between the *DBP* SNPs rs4588 and rs7041 and T1DM are of interest. This was reviewed by Penna-Martinez and Badenhoop who found that in the majority of the studies there was no relation between these SNPs and T1DM (66). As an example, Cooper et al. who included 720 cases and 2,610 controls and used a Mendelian randomization approach, found no relation between rs4588 and T1DM (67), whereas in the two studies that did find an association with rs7041 the total number of cases was only 154 (68, 69).

There are a few cross-sectional reports on serum DBP levels in patients with T1DM. In a study by Blanton et al. that included 203 subjects with T1DM and 153 controls, the serum DBP levels were ~10% lower in the T1DM patients (70). A similar result was found by Thraikill et al. but they could for a large part ascribe this to increased urinary loss of DBP in the urine (11). Low serum DBP levels have also been described in diabetic BB rats together with low serum 1,25(OH)_2_D levels accompanied with reduced duodenal calcium absorption, indicating the possible physiological importance of urinary DBP loss (71).

## Conclusions

For preventing or treating diabetes, the majority of clinical studies do not indicate a major role for vitamin D supplementation, with a possible exception for T1DM in children. As for many other presumed extra-skeletal effects of vitamin D, the effect on glucose metabolism must be small (if present at all) and accordingly difficult to demonstrate. In most of the vitamin D RCTs the results are also hampered by the inclusion of subjects who are not truly vitamin D deficient (72). However, since such subjects (and in particular young children) need vitamin D for bone health, there are many ethical problems in including vitamin D deficient subjects in long lasting RCTs. The “perfect” vitamin D RCT will therefore probably not be performed.

However, regarding vitamin D and health, the two crucial questions are how much vitamin D we need for skeletal health (which everyone agrees is vitamin D dependent), and if supplementation above that will give any additional health benefits.

So far, there are too few studies on the relative importance of measuring total or free 25(OH)D in diabetes and glucose metabolism, and too few studies on the importance of DBP concentration on development and progression of diabetes, to draw firm conclusion. However, since it is difficult to show an effect of vitamin D supplementation regarding diabetes, it follows that finding the right form or metabolite of vitamin D to measure (7), may for diabetes simply be a search for another biomarker (73). In disease states with clearly altered DBP levels, like pregnancy and liver cirrhosis, the situation obviously is different (9).

## Author Contributions

The author confirms being the sole contributor of this work and approved it for publication.

### Conflict of Interest Statement

The author declares that the research was conducted in the absence of any commercial or financial relationships that could be construed as a potential conflict of interest.
